# 

**Published:** 1889-03-23

**Authors:** 


### OUR NEW OFFICES,
### 139 AND 140, SALISBURY COURT, FLEET STREET, E.C.

Our readers are asked to note that with the new volume (which begins on April 6th) portraits of leaders in the medical, philanthropic and nursing world will be issued with the monthly parts. For the convenience of weekly subscribers, these portraits will be issued separately, at 3d. each, but must be ordered at an early date to secure prompt delivery on date of publication. In consequence of the growing requirements of The Hospital, and of the inconvenience to the trade arising from the distance of our present offices from the publishing centre,

**ON AND AFTER APRIL 1st**

The Hospital will be issued from, and all advertising and other communications should be sent to Our New Offices, 139 and 140, Salisbury Court, Fleet Street, E.C.

### Scraps and Gleanings.

Hospital Sunday Fund at Burton exceeded £200.

Dr. Wickens has been appointed Medical Officer of Ballarat, Victoria.

A death under chloroform lately occourred at Tavistock Cottage Hospital.

Swansea Provident Dispensary has over one thousand members on its books.

Mrs. Griffith Llewellyn has sent a cheque for £5,000 to the Swansea Hospital.

Wolverhampton Hospital Saturday Committee collected £2,748 last year; expenses, £59.

The amalgamation of Sunderland Infirmary and Children's Hospital has been carried out.

The Convalescent Home, Stillorgan, gave 700 patients the benefit of country air last year.

Mr. Rushton Parker has been elected honorary surgeon of Liverpool Royal Infirmary.

The town of Leith pays three shillings a-day for each fever patient sent to the Leith Hospital.

Viscount Torrington presided at the annual meeting of the Kent County Ophthalmic Hospital.

The Miners' Hospital, Redruth, owes its treasurer £47, a state of things which ought not to be.

The Dublin Hospital Sunday Fund for this year exceeds £4,000. The Earl of Meath subscribed £50.

The Royal Medical and Chirurgical Society is going to remove to new premises at 20, Hanover Square.

Mr. Robert Lang has presented six pictures by Bristol artists to the Bristol Sick Children's Hospital.

Wolverhampton General Hospital treated nearly 15,000 persons last year, at an average cost of 10s. each.

Mr. John Holm, F.R.C.S., has been appointed examiner in physical education to the London School Board.

Hockley Provident Dispensary admitted 500 new members last year, and has a balance of £62 in hand.

Loughborough Infirmary treated 80 in-patients last year, and over 1,000 out-patients. Total expenditure, £869.

The president of Queen's College, Belfast, has written a letter to the *Times* contradicting the statement of Archbishop Walsh that that college was not successful as a medical school.

At the late festival dinner in aid of Earlswood Asylum, the secretary announced subscriptions amounting to £1,865.

The committee of management of Ballymena have most lively meetings. They will discuss a mistake on a postcard for two hours.

At the Midsummer Quarter Sessions at Leeds, the dispute about closing Thoresby Street to enlarge the infirmary will be settled.

A successfull ball in aid of the Women's Hospital was held at Nottingham on March 1st. The Duchess of Rutland was a lady patroness.

Dundee Royal Infirmary has come in for three legacies lately. A nice sum should accrue to them also from Hospital Sunday, held on March 16th.

Liverpool Foundling Hospital is going to try the boarding-out plan, which has lately been worked with success by the London Foundling Hospital.

The inquiry into the amount of stimulants ordered for patients at Hull Royal Infirmary has proved that there is no excessive use of either wines or spirits.

Mr. Henry Smith, of King's College Hospital, has been presented with a piece of silver plate on his retirement from the chair of surgery of that hospital.

The Hospital Saturday Fund has received 100 guineas from the trustees of the Prisons Charities, on behalf of the Morley Seaside Convalescent Home.

Miss Isabella L. Bird (Mrs. Bishop) has started on a new journey, the most adventurous, it is said, of any she has undertaken. She is going in the interests of medical missions.

At the late annual meeting of governors of Queen's Hospital, Birmingham, great regret was expressed at the death of Miss Ryland, who was always a generous friend to that institution.

The Aberdeen Dispensary is in a most flourishing condition. The medical students of the Aberdeen Infirmary take advantage of the many good cases to be seen at the dispensary.

A meeting of the working men governors of the Royal Infirmary, Newcastle, was held in the library of the institution lately, for the purpose of electing nine of their number to the House Committee.

The Duncan Memorial Scheme has held a meeting and approved the proposed ambulance school at Woolwich; bust of Colonel Duncan for Holborn; and a memorial brass, which will fitly serve as a reminder of a noble life.

The poor of East London have lost a friend by the death of the Rev. Maurice Stack, M.A., Vicar of St. John's, Isle of Dogs. He was an earnest practical labourer in the promotion of everything tending to social amelioration.

A shocking accident lately occurred at a fencing club in Vienna. A fencer, making a heavy lunge, smashed his adversary's wire mask, and drove the foil completely through his eye into the brain, killing him almost instantaneously.

It is notified from the Lord Chamberlain's office that the Queen has been pleased to allow the members of the Order of the Hospital of St. John of Jerusalem in England to wear generally the insignia of their respective grades in the said Order.

News has been received from Cairo that the Khedive has made a donation of £50 to the Duncan Memorial Fund. It is also reported that many native officers of the Egyptian army are desirous of contributing to the fund, and that English officers in Egypt are interesting themselves in the work.

### Amusements and Relaxation.

### NEW PUZZLE PRIZE SCHEME.

SECOND QUARTERLY COMPETITION COMMENCED JANUARY 5TH, ENDS MARCH 30TH, 1889.

1. I rizes will be awarded for the best Original Puzzles in Prose or Verse relating to any of the Social, Political, or Philanthropical topics of the day. Competitors are requested to *strictly* observe this rule, or their contributions will be discredited.
2. Puzzles may take any form which the ingenuity of the competitors suggest. Answers must accompany contributions, and the source of obscure or ambiguous terms be given.
3. No competitor will be allowed to take more than one prize during the Quarter.
4. Eight Prizes of Standard Books will be given, the choice of the book to be left to the winner. First, Second, Third, Fourth, and Fifth Prizes for best Original Puzzles, value £1 1s., 15s., 10s. 6d., 7s. 6d., and 5s. each.
5. First, Second, and Third Prize for Solutions of 15s., 10s. 6d., and 5s. each.

N.B.—All contributions must reach the office, 38, Old Jewry, E.C., not later than the *First Post* on *Thursday* in each week.

### ORIGINAL PUZZLES FOR SOLUTION.

**Twelfth Week.**

No. 25. Double Acrostic.

We listen and rule,
Compare and discern,
And to king or to fool,
Act with honesty stern.

1. If ever in me you enter one day,
The sight of my horrors will drive you away!
2. The end of all things, the beginning of none,
When I am over, the tragedy's done.
3. Though very small, many in me you find,
Of every shape, and of every kind.
4. The colour of the sky, but of a darker shade,
Rarely to be seen, and never to be made.
5. The traveller sights me with feelings of joy,
Though sometimes a false hope his heart may decoy.
6. Once in Great Britain I reigned supreme,
But now, alas! am never seen.
7. If knowledge you want, then come to me quick,
For languages, science, houses, or music.

Morico.

No. 26. Original Anagrams.

1. Irish calls—One moment! P. 1

2. I thin each fine man.

Tinie.

**Marks for Original Puzzles.**

| Names. | No. of Puzzles sent in Mar. 14. | No. of Marks Mar. 14. | Totals. | Names. | No. of Puzzles sent in Mar. 14. | No. of Marks Mar. 14. | Totals. |
|---|---|---|---|---|---|---|---|
| Cotonopolis... | 1 | 3 | 43 | Prior........... | 3 | 3 | 22 |
| Tinie........... | 3 | 6 | 50 | Lightowlers . | 3 | 3 | 27 |
| Jenny Wren . | — | — | 4 | Twentieth ... | 1 | 3 | 6 |
| Patience ...... | 3 | 5 | 58 | Crocodile...... | 2 | 6 | 14 |
| Padre ......... | — | — | 3 | Nabob ......... | 1 | 2 | 13 |

**Answers.**

No. 21. Double Acrostic.

M u D
U lste R
S ciatic A
I sla M
C hin A

No. 22. Charade. Par-nell=Parnell.

**Marks for Solution of Puzzles.**

Total Marks.—Flora (1), 10; Mater (1), 8; Scrooge (1), 10; Patience 4; Lady Clara (2), 11; Ruffles (1), 7; Lauriston, 1; Reldas (2), 8; Jenny Wren, 4; Condorcet 2; Tinie, 2.

[*The Figures in parentheses are Marks for the week ending March 14th.*]

### SPECIAL NOTICE.

A New Orginal Acrostic Competition will be commenced in these columns the first week in April.

Acrostics to be sent in every fortnight, commencing Thursday, April 4th, and ending June 27th.

Two prizes will be given—First Prize of One Guinea, and Second Prize of 10s. 6d.

Acrostics will be credited according to merit, six marks being the highest score given for any one Acrostic.

In addition to the above prizes, one of *Half-a-Guinea* will be given for Solution of Acrostics set.

One mark only will be given for the correct solution of all lights of each Acrostic.

**Word Competition.**

At the request of many of our readers we have decided to start another weekly Word Dissection, commencing April 4th, and ending July 4th.

Three Prizes of 15s., 10s., 5s., will be given for the largest number of words derived from the word set for dissection.

Proper names, abbreviations, foreign words, words of less than four letters, and repetitions are barred; plurals, and past and present participles of verbs, are allowed. Nuttall's *Standard* dictionary only to be used.

N.B.—All words sent in must be arranged *alphabetically*, with correct total affixed.

(For Conditions see last week's The Hospital.)

